# Slower-X: reduced efficiency of selection in the early stages of X chromosome evolution

**DOI:** 10.1093/evlett/qrac004

**Published:** 2023-01-31

**Authors:** Andrea Mrnjavac, Ksenia A Khudiakova, Nicholas H Barton, Beatriz Vicoso

**Affiliations:** Institute of Science and Technology Austria, Am Campus 1, 3400 Klosterneuburg, Austria; Institute of Science and Technology Austria, Am Campus 1, 3400 Klosterneuburg, Austria; Institute of Science and Technology Austria, Am Campus 1, 3400 Klosterneuburg, Austria; Institute of Science and Technology Austria, Am Campus 1, 3400 Klosterneuburg, Austria

**Keywords:** sheltering, faster-X theory, X chromosome, young sex chromosomes, degeneration, adaptation, demasculinization

## Abstract

Differentiated X chromosomes are expected to have higher rates of adaptive divergence than autosomes, if new beneficial mutations are recessive (the “faster-X effect”), largely because these mutations are immediately exposed to selection in males. The evolution of X chromosomes after they stop recombining in males, but before they become hemizygous, has not been well explored theoretically. We use the diffusion approximation to infer substitution rates of beneficial and deleterious mutations under such a scenario. Our results show that selection is less efficient on diploid X loci than on autosomal and hemizygous X loci under a wide range of parameters. This “slower-X” effect is stronger for genes affecting primarily (or only) male fitness, and for sexually antagonistic genes. These unusual dynamics suggest that some of the peculiar features of X chromosomes, such as the differential accumulation of genes with sex-specific functions, may start arising earlier than previously appreciated.

## Introduction

In many species, the sex of an individual is determined by a pair of sex chromosomes, such as the X and Y of mammals, or Z and W in the case of the female heterogametic system (Bachtrog et al., 2014). Although we focus on the more commonly studied XY case, the models discussed here also apply to ZW systems (by switching the sexes). Sex chromosomes arise from a pair of autosomes when one of them acquires a sex-determining gene (Vicoso, 2019; Wright et al., 2016). This is often coupled with the suppression of recombination between X and Y chromosomes in the region surrounding the sex-determining gene. Recombination suppression can spread progressively along the chromosomes in a stepwise manner, creating distinct “evolutionary strata,” i.e., regions that stopped recombining at the same time, and which consequently have different levels of XY divergence (Jefferies et al., 2021; Ponnikas et al., 2018). Recombination suppression between X and the Y reduces the efficiency of selection on the Y chromosome, leading to progressive gene loss on the Y (Bachtrog, 2013; Engelstädter, 2008). The resulting imbalance in gene copy number in males drives the evolution of dosage compensation mechanisms, which results in equal expression levels in males and females in somatic tissues. In male gonads, on the other hand, X chromosomes are often downregulated or completely inactivated (Larson et al., 2018; Mahadevaraju et al., 2021). In addition to this unusual regulatory architecture, X chromosomes have been found to differ from autosomes in various ways. One consistent feature is the over- and under-representation of genes with sex-specific patterns of expression (sex-biased genes), although the specific direction of the enrichment varies across species. For instance, the Drosophila X chromosome has a deficit of male-biased genes, whereas the mammalian X is enriched for genes with male-specific functions (Gurbich & Bachtrog, 2008). X chromosomes also often have more transposable elements and repeats and have different gene densities, than autosomes. Finally, genes move out of X chromosomes more often than expected in both mammals and Drosophila (Gurbich & Bachtrog, 2008). Understanding what evolutionary processes drive these patterns has been the goal of extensive theoretical and empirical research (Charlesworth et al., 2018; Gurbich & Bachtrog, 2008; Meisel & Connallon, 2013; Rice, 1984; Vicoso & Charlesworth, 2006).

Since males have only one X chromosome, whereas females have two, X chromosomes differ from autosomes in key population parameters. In a population with equal sex ratio and *N* individuals, there are 1.5*N* X chromosomes and *2N* sets of autosomes, so the population size of an X chromosome is three-quarters of the population size of an autosome. Furthermore, X chromosomes are transmitted two-thirds of the time through females and one-third of the time through males, whereas autosomes spend an equal amount of time in males and females. Finally, once Y-linked genes have been lost, recessive mutations arising on an X chromosome are immediately selected in hemizygous males. How these peculiarities affect the evolutionary dynamics of X-linked loci has been previously modeled (Charlesworth et al., 1987; Hitchcock & Gardner, 2020; Meisel & Connallon, 2013; Patten, 2019; Rice, 1984; Vicoso & Charlesworth, 2009). Rice (1984) found that new recessive, male-beneficial sexually antagonistic mutations (i.e., mutations with opposite fitness effects in males and females) can invade a population more easily if they are X-linked than autosomal, and suggested that this may lead to an excess of X-linked genes underlying sexual dimorphism. However, this prediction depends on the dominance coefficient of sexually antagonistic mutations (Rice, 1984), for which we have little empirical evidence, so it is hard to make clear predictions as to whether the X or autosomes are more favorable to the invasion of sexually antagonistic mutations (Ruzicka & Connallon, 2020). Charlesworth et al. (1987) further showed that selection on recessive mutations is stronger on the X chromosome than on the autosomes, resulting in faster substitution rates of recessive beneficial mutations on the X chromosome than on the autosomes, a pattern known as the “faster-X effect” (Vicoso & Charlesworth, 2006, 2009). Conversely, they found that slower substitution rates are expected on X-linked loci for deleterious recessive mutations. Mutations with stronger effects on male than female fitness are particularly prone to faster-X evolution (whereas mutations with female-limited effects are exempt). Extensions of this theory have further shown that male-biased mutation rates (Kirkpatrick & Hall, 2004) and increased variance in male relative to female reproductive success (through its effect on X and autosome effective population size) can increase the faster-X effect (Vicoso & Charlesworth, 2009). On the other hand, if positive selection acts on standing variation rather than new mutations, faster-X evolution is not expected (Orr & Betancourt, 2001). Several studies have attempted to detect a faster-X effect empirically, for instance by testing for a higher proportion of adaptive substitutions on the X chromosome compared to autosomes. Faster-X divergence and faster-X adaptation have been found in various vertebrate and invertebrate clades (Bechsgaard et al., 2019; Charlesworth et al., 2018; Llopart, 2018; Meisel & Connallon, 2013; Mongue et al., 2022; Rupp et al., 2017; Sackton et al., 2014), but not all (Pinharanda et al., 2019, Rousselle et al., 2016; Radhakrishnan & Valenzuela, 2017). Similarly, an excess of sexually antagonistic effects has been suggested in some studies (Abbott et al., 2020; Foerster et al., 2007; Gibson et al, 2002; Innocenti & Morrow, 2010) but not others (Fry, 2010; Ruzicka et al., 2019; Ruzicka & Connallon, 2022). This is further complicated by the fact that the quantitative genetic measures typically used to detect sexual antagonism are biased toward the detection of X-linked effects (Ruzicka & Connallon, 2020). Because these theories predict different adaptive trajectories for the X and autosomes, they have been invoked to account for various unusual patterns observed on X chromosomes, such as the differential representation of genes with sex-biased expression or the excess movement out of the X (reviewed in Gurbich & Bachtrog, 2008; Vicoso & Charlesworth, 2006).

Much less is known about the evolution of X-linked genes during the early stages of sex chromosome evolution, when the majority of genes on the Y chromosome are functional, but recombination between X and the Y is suppressed. The evolution of X-linked genes under such a scenario (in which there is no recombination between homologous loci on the X and Y, but both X and Y homologs are functional and affect fitness), has not been theoretically explored (but see Engelstaedter, 2008, who modeled how the accumulation of deleterious mutations on the X affects Y-chromosome evolution). By contrast, several empirical studies recognized that young, diploid X-linked loci can have unusual evolutionary dynamics (Nozawa et al., 2016, 2021; Wright et al., 2017). Faster rates of nonsynonymous substitutions and enrichment of sex-biased genes have been detected on young and undifferentiated X/Z chromosomes (Pucholt et al., 2017; Wright et al., 2017). Furthermore, Nozawa et al. (2016, 2021) found evidence of accelerated pseudogenization rates on the young X chromosomes of several Drosophila lineages, compared with both the ancient X chromosome and the autosomes. They hypothesized that mechanisms similar to the ones causing degeneration of the Y chromosome could be driving the degeneration of the X chromosome: first, the X chromosome has a smaller population size compared to the autosomes, which makes selection less efficient. Second, since X chromosomes do not recombine in males, their effective population size can be further reduced. However, the opposite is expected in *Drosophila*, as in this clade recombination is restricted to females, where X chromosomes are found two third of the time. Third, female-biased transmission could be driving the loss of genes that are unimportant for females. Another effect that could contribute to the accumulation of deleterious mutations on young X-linked genes is sheltering by the functional gene copy on the Y chromosome. New mutations arising on a diploid X-locus are always heterozygous in males because there is no recombination between the X and the Y, and their phenotypic effect is masked by the ancestral allele on the Y. While the role of sheltering has been appreciated in other contexts, such as the degeneration of Y chromosomes (Bachtrog, 2013; Muller, 1914; Nei, 1970) and the evolution of recombination suppression (Antonovics & Abrams, 2004; Charlesworth & Wall, 1999; Jay et al., 2022; Olito et al., 2022), it is unclear to what extent it affects early X-chromosome evolution. Here, we model evolutionary rates of X-linked loci with functional Y copies under various selective regimes, to formally explore how these different processes shape the early stages of X chromosome evolution.

## Methods

### The diffusion approximation

Substitution rates can be calculated as the average number of mutations entering a population in one generation times the fixation probability of those mutations (Kimura & Ohta, 1971). To derive probabilities of fixation for autosomal and hemizygous X-linked loci, Vicoso and Charlesworth (2009) used the diffusion approximation. Here, we extend their model to diploid X-linked loci.

Let A_1_ and A_2_ be alleles for some locus, with frequencies *(1*−*p)* and *p*, respectively, and fitness effects as noted in Table 1.

**Table 1.** Relative fitnesses in females and males for autosomal, hemizygous X-linked, and diploid X-linked loci.

		**Females**			**Males**		
*Autosomal*							
	Genotypes	A_1_A_1_	A_1_A_2_	A_2_A_2_	A_1_A_1_	A_1_A_2_	A_2_A_2_
	Fitness	*1*	*1+hs*_*f*_	*1+s*_*f*_	*1*	*1+hs*_*m*_	*1+s*_*m*_
*Hemizygous X-linked*							
	Genotypes	A_x1_A_x1_	A_x1_A_x2_	A_x2_A_x2_	A_x1_	A_x2_	
	Fitness	*1*	*1+hs*_*f*_	*1+s*_*f*_	*1*	*1+s*_*m*_	
*Diploid X-linked*							
	Genotypes	A_x1_A_x1_	A_x1_A_x2_	A_x2_A_x2_	A_y1_A_x1_	A_y1_A_x2_	
	Fitness	*1*	*1+hs*_*f*_	*1+s*_*f*_	*1*	*1+hs*_*m*_	

We can estimate the fixation probability of allele A_2_ using the diffusion approximation (Ewens, 2004). The fixation probability of an allele with the initial frequency *p* is given by the function *U(p)*:


$$\begin{array}{l} U(p)=\frac{\overset{p}{\underset{0}{\int }}G(y)dy}{\overset{1}{\underset{0}{\int }}G(y)dy}, \end{array}$$ (1)

With $G(y)=exp(-2\underset{0}{\overset{y}{\int }}\frac{M(x)}{V(x)}dx),$

where *M(x)* and *V(x)* are, respectively, the expectation and the variance of the change of allele frequency.

Assuming a weak effect of selection in each sex, so that second-order terms are small enough to be neglected, the fitness of a genotype can be approximated as the average of fitness effects in males and females (Charlesworth & Charlesworth, 2010, ch. 3.1). For a diploid X-linked locus, the expected change of an allele A_2_ frequency due to selection is given by:


$$\begin{array}{l} M_{dX}(x)\approx x(1-x)(\frac{2}{\text{3}}(w_{2f}-w_{1f})+\frac{1}{\text{3}}(w_{2m}-w_{1m})), \end{array}$$

where *x* is the frequency of the allele A_2_, w_1f_ and w_1m_ are marginal fitnesses of allele A_1_ in females and males, respectively, and w_2f_ and w_2m_ are marginal fitnesses of allele A_2_ in females and males respectively. Calculating these marginal fitnesses from Table 1, we have:


$$\begin{array}{l} M_{dX}(x)\approx x(1-x)(\frac{2}{\text{3}}s_{f}(h+x(1-2h))+\frac{1}{\text{3}}hs_{m}), \end{array}$$

where *h* is the dominance coefficient.

We now divide $M_{dX}(x)$ by the variance in the change of allele A_2_ frequency due to sampling drift,


$$\begin{array}{l} V_{dX}(x)\approx \frac{x(1-x)}{2N_{eX}}, \end{array}$$

where *N*_*eX*_ is the effective population size of the X chromosome, and integrate to find *G(y)*. This can be written in terms of mean selection averaged across sexes,

$\bar{s}=\;\frac{2}{\text{3}}\frac{s_{f}}{2}+\frac{1}{\text{3}}hs_{m}=\frac{s_{f}+hs_{m}}{3}$ , and $\sigma =\frac{s_{f}(1-2h)}{3}$, which is zero if there is no dominance (*h = 1/2*). Then:


$$\begin{array}{l} \begin{array}{ll} G_{dX}(y) & =exp(-2\underset{0}{\overset{y}{\int }}\frac{M(x)}{V(x)}dx)\\ & =exp(-\frac{4}{\text{3}}N_{eX}(2s_{f}+s_{m})h\;\;\;\underset{0}{\overset{y}{\int }}dx-\frac{8}{\text{3}}N_{eX}s_{f}(1-2h)\underset{0}{\overset{y}{\int }}xdx\\ & =exp(-\frac{4}{\text{3}}N_{eX}(2s_{f}+s_{m})hy-\frac{4}{\text{3}}N_{eX}s_{f}(1-2h)y^{2})\\ & =exp(-4N_{eX}(\frac{1}{\text{3}}(s_{f}+hs_{m})y-\frac{1}{\text{3}}s_{f}(1-2h)y(1-y)))\\ & =exp(-4N_{eX}(\;\;\;\bar{s}y-\sigma y(1-y))) \end{array} \end{array}$$ (2)

We are interested in the probability of fixation of a single new mutation, which is initially at $p=\frac{2}{3N}$; the substitution rate is 1.5NμU(p)=μU(p)/p, where μ is the mutation rate. Following Eq. (1), the fixation probability *U(p)* becomes:


$$\begin{array}{l} \begin{array}{ll} U(p) & =\frac{\overset{p}{\underset{0}{\int }}G(y)dy}{\overset{1}{\underset{0}{\int }}G(y)dy}\\ & =\frac{erf(\frac{N_{eX}(\;\;\;\bar{s}\;\;\;+\sigma (2p-1))}{\sqrt{N_{eX}\sigma }})-erf(\frac{N_{eX}(\;\;\;\bar{s}\;\;\;-\sigma )}{\sqrt{N_{eX}\sigma }})}{erf(\frac{N_{eX}(\;\;\;\bar{s}\;\;\;-\sigma )}{\sqrt{N_{eX}\sigma }})-erf(\frac{N_{eX}(\;\;\;\bar{s}\;\;\;-\sigma )}{\sqrt{N_{eX}\sigma }})\;\;\;}\\ & =\frac{\frac{4p\sqrt{N_{eX}\sigma }}{\sqrt{\pi }}exp(-\frac{N_{eX}{\left(\;\;\;\bar{s}-\sigma \right)}^{2}}{\sigma })}{erf(\frac{N_{eX}(\;\;\;\bar{s}\;\;\;-\sigma )}{\sqrt{N_{eX}\sigma }})-erf(\frac{N_{eX}(\;\;\;\bar{s}\;\;\;-\sigma )}{\sqrt{N_{eX}\sigma }})\;\;\;}+O(p^{2}), \end{array} \end{array}$$

where $O(p^{2})$ are higher order terms in the Taylor series when *p* is close to zero. Assuming weak selection and sufficiently large effective population size, $O(p^{2})$ can be neglected for new mutations. Then, the first-order approximation in *p* for the substitution rate is:


$$\begin{array}{l} \mu (U(p)/p)=4\mu \sqrt{\frac{N_{eX}\sigma }{\pi }}\;\;\;\frac{exp(-N_{eX}{\left(\;\;\;\bar{s}-\sigma \right)}^{2}/\sigma )}{\left(erf(N_{eX}(\;\;\;\bar{s}+\sigma )/\sqrt{N_{eX}\sigma })-erf(N_{eX}(\;\;\;\bar{s}-\sigma )/\sqrt{N_{eX}\sigma })\right)} \end{array}$$ (3)

Note that the substitution rate relative to mutation depends only on $N_{eX}\bar{s}$ and $N_{eX}\sigma$; in the additive case (*h* = 1/2), it simplifies to Kimura’s formula, $4N_{eX}\;\;\;\bar{s}/(1-exp(-4N_{eX}\;\;\;\bar{s}))$.

Also, note from Table 1 that the only difference between the hemizygous and the diploid X cases is that *s*_*m*_ is replaced by *hs*_*m*_ in the latter case; thus, the results of Vicoso and Charlesworth (2009) for the hemizygous case can be found from the results given here simply by replacing *s*_*m*_ by *s*_*m*_*/h.*

We implemented the numerical integration in R (*version 4.1.1*, code available at https://github.com/andrea-mrnjavac/X-chromosome-theory/blob/main/fixation_probability_functions.R), which allowed us to derive substitution rates for autosomal, hemizygous X-linked and diploid X-linked loci over the range of dominance coefficients and selective effects in males and females. A GUI application with implemented fixation probability functions for autosomal, hemizygous X-linked, and diploid X-linked loci is available at *https://degenerate-x.science.ista.ac.at/*, and allows the user to explore the difference among substitution rates of autosomal, hemizygous X-linked and diploid X-linked loci over the range of parameter values (selective effects in males and females and dominance coefficient), assuming effective population size of an X chromosome is three fourth of autosomal effective population size. In addition, we modeled evolutionary rates on X chromosomes and autosomes assuming equal population sizes for autosomes and X chromosomes, in order to disentangle the effects of effective population size and sheltering.

### Branching process approximation

To model the substitution rates of strongly beneficial mutations at diploid X-linked loci with functional but non-recombining Y gametolog and allow comparisons with the classic Charlesworth et al. (1987) result, we used Haldane’s branching process approximation (Charlesworth et al., 1987, 2018; Haldane, 1927; Meisel & Connallon, 2013; Vicoso & Charlesworth, 2006).

If *N*_*e*_*sh* is sufficiently large (1*/N*_*e*_*<< sh <<* 1), the fixation probability of a new beneficial mutation can be approximated as twice the advantage of a heterozygous genotype (Haldane, 1927).

Taking into account the effects of a mutation in males and females separately (Vicoso & Charlesworth, 2006), the fixation probability of a single new beneficial mutation at an autosomal locus can be approximated as:


$$\begin{array}{l} P_{A}(\frac{1}{2N})\approx \frac{1}{\text{2}}2hs_{f}+\frac{1}{\text{2}}2hs_{m}\approx h(s_{f}+s_{m}), \end{array}$$

where s_f_ and s_m_ are selection coefficients in females and males, respectively, *h* is the dominance coefficient, and *N* is the number of diploid individuals, as before.

For diploid X-linked loci, the fixation probability of a new mutation is then:


$$\begin{array}{l} P_{X}(\frac{1}{1.5N})\approx \frac{2}{\text{3}}2hs_{f}+\frac{1}{\text{3}}2hs_{m}\approx \frac{2}{\text{3}}h(2s_{f}+s_{m}). \end{array}$$

If we assume the number of X chromosomes in a population is three fourth the number of autosomes, and dominance is the same in males and females, as well as the mutation rate, the following substitution rates are then:

$K_{A}\approx 2N\mu h(s_{f}+s_{m})$ for autosomal loci, and

$K_{X}\approx \frac{3}{\text{2}}N\mu \frac{2}{\text{3}}h(2s_{f}+s_{m})\approx N\mu h(2s_{f}+s_{m})$ for diploid X loci.

The ratio of diploid X to autosomal adaptive substitution rates is:


$$\begin{array}{l} R\approx \frac{N\mu h(2s_{f}+s_{m})}{2N\mu h\left(s_{f}+s_{m}\right)}\approx \frac{2s_{f}+s_{m}}{2s_{f}+2s_{m}} \end{array}$$ (4)

## Results

### Diploid X loci adapt slower and accumulate deleterious mutations faster

In addition to previously derived substitution rates on hemizygous X loci and autosomal loci (Charlesworth et al., 1987; Vicoso & Charlesworth, 2009), we used the diffusion approximation to estimate fixation probabilities of new mutations arising at diploid X-linked loci (which have a functional homolog on the Y), and their substitution rates.

**Figure 1.**
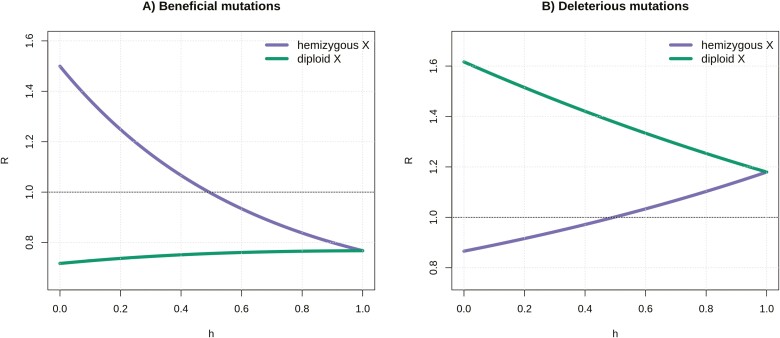
Ratios of substitution rates (*R*) on hemizygous X to autosomal loci and diploid X to autosomal loci as functions of dominance coefficient *h*, for beneficial, *N*_*eA*_*s* = 3 (A) and deleterious, *N*_*eA*_*s* = −1 mutations (B).

We verified these estimates with individual-based forward genetic simulations in SLiM (Haller & Messer, 2019) (Supplementary Figure 1, details of simulations are given in Supplementary Material). The X/A ratios of substitution rates (*R*) for beneficial and deleterious mutations and for diploid and hemizygous X-linked loci are visualized in Figure 1. We recover the previously described faster-X effect for hemizygous X-linked loci (Charlesworth et al., 1987; Vicoso & Charlesworth, 2006, 2009), where recessive beneficial mutations accumulate faster on the hemizygous X loci, while dominant beneficial mutations accumulate faster on the autosomes compared to the hemizygous X loci. On the contrary, diploid X loci exhibit a slower-X effect regardless of the dominance coefficient: the substitution rate of beneficial mutations is lower at diploid X loci compared to autosomal and hemizygous X loci, in agreement with reduced efficiency of selection on diploid X loci due to sheltering and reduced effective population size (Table 1). Substitution rates of deleterious mutations follow the opposite pattern, with diploid X loci accumulating deleterious mutations faster than autosomal and hemizygous X loci. These results, therefore, show that young diploid X-linked genes have reduced adaptive potential, and increased accumulation of deleterious mutations, relative to both hemizygous X-linked genes and autosomal genes.

To disentangle the effects of reduced effective population size and sheltering on substitution rates of diploid X loci, we calculated the ratio of substitution rates in the case where *N*_*eX*_ = *N*_*eA*_, as is approximately the case in *Drosophila melanogaster* due to the effect of selection at linked sites (although differences in male and female fitness variance may also play a role (Charlesworth, 2001)): since there is no recombination in males, X chromosomes have higher effective recombination rates than autosomes, which increases the *N*_*eX*_/*N*_*eA*_ ratio (Charlesworth et al., 2018). The results show that in this case there is still a “slower-X” effect for diploid X loci, but only for recessive mutations (Supplementary Figure 4).

As an example, Figure 1 shows the ratios of substitution rates (*R*) for beneficial mutations with *N*_*eA*_*s* = 3, and for deleterious mutations with *N*_*eA*_*s* = −1, where equal effects in males and females are assumed (s = s_f_ = s_m_), but a wide range of positive and negative *N*_*eA*_*s* values (we modeled *N*_*eA*_*s* values from −3 to 5) yield the same qualitative pattern. The effect of different *N*_*eA*_*s* values of a mutation on the X/A ratio of substitution rates can be explored in a GUI web application provided at *https://degenerate-x.science.ista.ac.at/*. However, it is worth noting that the ratio of substitution rates at diploid X loci compared to autosomal loci (*R*) increases exponentially with the strength of the deleterious effect of a mutation (Supplementary Figure 2). This means that mutations with stronger deleterious effects will be fixed much more often in diploid X loci relative to an autosome.

While the original faster-X publication (Charlesworth et al., 1987) focused on R (in that case the autosome:X substitution rate ratio), evolutionary rates are usually measured as the rate of nonsynonymous substitutions (*dN*), normalized by the synonymous rate of substitutions (*dN*/*dS*, with *dS* acting as a proxy for neutral divergence) (Meisel & Connallon, 2013). To facilitate comparisons with such data, we also plot the substitution rate of beneficial and deleterious mutations after normalizing them by the neutral substitution rate (qualitative patterns remain the same, Supplementary Figure 3).

Similar results are recovered with the branching process approximation (see methods). Equation (4) shows that for strongly beneficial mutations (where *N*_*e*_*sh* is sufficiently large), the X/A ratio of substitution rates does not depend on the dominance coefficient at diploid X loci, in contrast with the previously described X/A ratio of adaptive evolutionary rates at hemizygous X loci, which exhibit faster-X effect for recessive mutations. More precisely, our results show that for mutations with female-limited selective effects, substitution rates are the same at autosomal and diploid X loci, that is, *R* = 1; for mutations with male-limited selective effects, *R* = 1/2, that is, adaptation rate on diploid X is half the adaptation rate on autosomes, and for mutations with equal effects in males and females, *R* = 3/4. These results indicate a slower-X effect for all strongly beneficial mutations arising on diploid X loci and having an effect in males.

### The “slower X” effect is strongest for male-biased mutations

**Figure 2.**
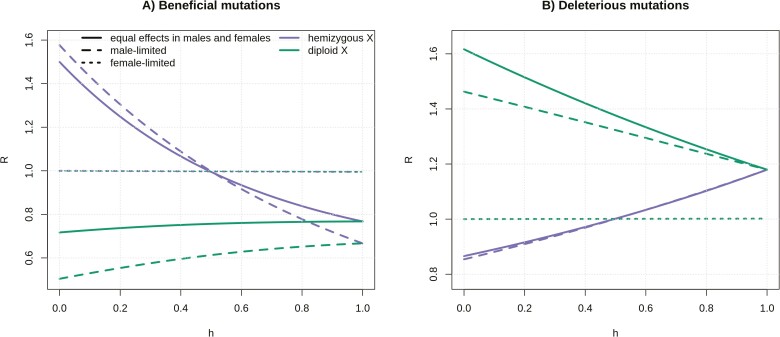
Ratios of X/A substitution rates (*R*) of male-limited mutations (*N*_*e*_*s*_*m*_ = 3, *N*_*e*_*s*_*f*_ = 0 for and *N*_*e*_*s*_*m*_ = −1, *N*_*e*_*s*_*f*_ = 0 for beneficial and deleterious mutations respectively) are plotted alongside ratios of substitution rates of mutations with equal effects (*N*_*e*_*s*_*m*_ = 3, *N*_*e*_*s*_*f*_ = 3 for and *N*_*e*_*s*_*m*_ = −1, *N*_*e*_*s*_*f*_ = −1 for beneficial and deleterious mutations respectively), and female-limited (*N*_*e*_*s*_*m*_ = 0, *N*_*e*_*s*_*f*_ = 3 for and *N*_*e*_*s*_*m*_ = 0, *N*_*e*_*s*_*f*_ = −1 for beneficial and deleterious mutations, respectively) effects for beneficial (A) and deleterious (B) mutations, as a function of dominance coefficient, *h. R* for female-biased mutations is between *R* for female-limited mutations and *R* for mutations with equal effects in males and females, while *R* for male-biased mutations is between *R* for male-limited mutations and *R* for mutations with equal effects in both sexes.

We also aimed to disentangle how selective effects in males and females separately affect the substitution rate at diploid X loci. Mutations can have different effects in males and females: they can be sex-limited, affecting only the fitness of one sex, sex-biased, if they have a stronger effect on the fitness of one sex than the other, or sexually antagonistic, if they have fitness effects of opposite signs in the two sexes. We can intuitively see from the fitness table (Table 1) that the differences in evolutionary rates of diploid X, hemizygous X, and autosomal loci result from the differences in the male part of the fitness table. Indeed, similar to Charlesworth et al. (1987), we find that evolutionary rates are the same at autosomal, hemizygous X-linked, and diploid X-linked loci for female-limited mutations (mutations affecting only female fitness, e.g., *N*_*e*_*s*_*m*_ = 0, *N*_*e*_*s*_*f*_ = 3 in Figure 2A, or *N*_*e*_*s*_*m*_ = 0, *N*_*e*_*s*_*f*_ = −1 in Figure 2B). On the other hand, the X/A ratio of substitution rates (*R*) for beneficial male-limited mutations (*N*_*e*_*s*_*m*_ = 3, *N*_*e*_*s*_*f*_ = 0), and to a smaller extent male-biased mutations, is lower than R for mutations with an equal effect in both males and females (*N*_*e*_*s*_*m*_ = 3, *N*_*e*_*s*_*f*_ = 3) (Figure 2A). Male-limited and male-biased mutations are primarily under selection in males, where their effect is masked by the ancestral allele on the Y, resulting in a stronger “slower-X effect.” Analogously, deleterious male-limited and male-biased mutations accumulate faster at diploid X loci than at autosomal loci, and faster than deleterious female-limited and female-biased loci with the corresponding fitness effects (e.g., comparing male-biased mutations with *N*_*e*_*s*_*m*_ = −1, *N*_*e*_*s*_*f*_ = −0.5 to female-biased mutations with *N*_*e*_*s*_*m*_ = −0.5, *N*_*e*_*s*_*f*_ = −1). Counterintuitively, Figure 2B shows that *R* for a diploid X is larger for mutations with equal fitness effects in males and females than for male-limited and male-biased mutations. This is because mutations affecting both sexes (*N*_*e*_*s*_*m*_ = −1, *N*_*e*_*s*_*f*_ = −1) are overall more deleterious than mutations affecting males (*N*_*e*_*s*_*m*_ = −1, *N*_*e*_*s*_*f*_ = 0) or females only, and R for the diploid X increases exponentially with the strength of the deleterious effect of a mutation (Supplementary Figure 2). To summarize, the X/A ratio of substitution rates for deleterious mutations at diploid X will be greater than 1 as long as the mutation has an effect in males, and mutations with a stronger deleterious effect in males than in females will accumulate in excess on the young X compared to autosomes.

**Figure 3.**
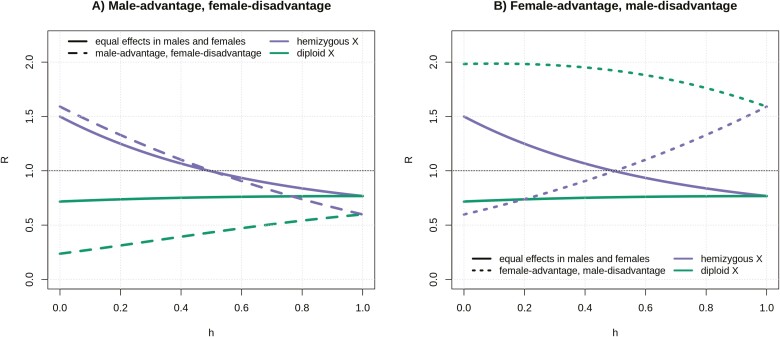
Ratios of X/A substitution rates (*R*) for sexually antagonistic mutations carrying male advantage and female disadvantage (*N*_*e*_*s*_*m*_ = 3, *N*_*e*_*s*_*f*_ = −3) (A) and mutations carrying female advantage and male disadvantage *N*_*e*_*s*_*m*_ = −3, *N*_*e*_*s*_*f*_ = 3 (B) plotted along the *R* for mutations with equal effects in males and females, as a function of dominance coefficient, *h*.

Sexually antagonistic mutations are expected to accumulate faster on an ancient hemizygous X than on the autosomes if they are recessive and male-beneficial, or dominant and female-beneficial (Rice, 1984; Vicoso & Charlesworth, 2009, and Figure 3). One assumption of these studies, which we also make here, is that the dominance coefficient of antagonistic mutations is the same in males and females (see Fry, 2010, for results when this does not hold). The resulting differential accumulation of sexually antagonistic mutations has been proposed to influence the evolution of sex chromosomes and their role in encoding sexual dimorphism (Fry, 2010; Rice, 1984). Figure 3 shows that mutations providing an advantage to males but a disadvantage to females (*N*_*e*_*s*_*m*_ = 3, *N*_*e*_*s*_*f*_ = −3) accumulate much more slowly at diploid X loci than at autosomal or hemizygous X loci, independent of their dominance coefficient. Mutations carrying female advantage and male disadvantage (*N*_*e*_*s*_*m*_ = −3, *N*_*e*_*s*_*f*_ = 3), on the other hand, accumulate faster at diploid X loci. Taken together, these results show that the reduced efficacy of selection on a diploid X is highly influenced by sex-specific fitness effects, with mutations that benefit primarily males tending to accumulate slower, and mutations that are detrimental to males accumulating faster whether they benefit females or not.

## Discussion

There has been extensive theoretical and empirical work on the “faster-X effect” expected on X chromosomes with a degenerated Y counterpart (reviewed in Charlesworth et al., 2018; Meisel & Connallon, 2013). Here, we modeled evolutionary rates of diploid X-linked loci, with a functional, but non-recombining, gametolog on the Y counterpart. Our results show very different evolutionary dynamics for diploid and hemizygous X loci. We make two key predictions for the evolution of young X-chromosomes: (a) Selection efficacy is reduced, such that fewer beneficial mutations and more deleterious mutations will fix there compared to an autosomal locus. (b) The relaxation of selection is stronger for mutations that primarily affect male fitness and/or benefit males at the expense of females. Over time, this may lead to the “demasculinization” of the young X chromosome, i.e., the degeneration of genes with male-important functions, and/or the failure to acquire new genes with male functions. This is in contrast to hemizygous X-linked loci on differentiated X chromosomes, which may exhibit faster adaptation and masculinization (as long as beneficial mutations are generally recessive).

These peculiar evolutionary dynamics of diploid X loci are caused by: (a) a smaller effective population size compared to autosomes, (b) female-biased transmission, and (c) sheltering of partly recessive X-linked mutations in males by an ancestral allele on the Y. By exploring a range of parameters, we could to some extent quantify the individual contribution of these effects. Sheltering does not affect fully dominant mutations, and the difference in *R* when *h* = 0 and *h* = 1 shows that it can have a substantial effect on rates of adaptive and maladaptive divergence (Figures 1–3). When the reduction in *N*_*e*_ is removed (*N*_*eA*_ = *N*_*eX*_), reduced efficacy of selection is only detected when mutations are at least partly recessive (due to sheltering, Supplementary Figure 4A and B) and/or have male-biased or male-limited effects (due to female-biased transmission and a stronger effect of sheltering, Supplementary Figure 4C and D). The relative importance of these effects depends on the strength of fitness effects in males, as sheltering only affects mutations expressed in this sex. Finally, both female-biased transmission and sheltering contribute to the proposed demasculinization, as male-important mutations are affected disproportionately (Supplementary Figure 4).

Only a few empirical studies have explicitly compared the evolution of diploid X-linked genes to autosomal control. Several of them investigated the young neo-X chromosome found in the *Drosophila miranda* lineage, where a pair of autosomes fused with the ancestral Y chromosome and became a neo-X and neo-Y a little over 1 million years ago. Around 40% of genes on the neo-Y are still functional (Nozawa et al., 2016; Zhou & Bachtrog, 2012). Zhou and Bachtrog (2012) found that hemizygous X loci adapt faster than diploid X loci on the neo-X chromosome in *D. miranda*. Nozawa et al. (2016) further detected evidence of an accelerated pseudogenization rate on the neo-X chromosome in *D. miranda* after it became X-linked. They compared the neo-X chromosome in *D. miranda* to the corresponding autosome in *D. pseudoobscura*, and found that genes that were ancestrally under reduced selective constraints (have higher *dN*/*dS* values in *D. pseudoobscura*) and genes with ancestrally male-biased function (approximated from *D. pseudoobscura* F/M expression ratios) were more likely to be pseudogenized on the neo-X than we would expect if it was an autosome. Their analyses suggest that the reduction in efficiency of selection, especially for male-biased genes, is causing the accelerated pseudogenization rate on the young X chromosome. Recently, Nozawa et al. (2021) found degeneration of neo-X chromosomes in two other *Drosophila* species with independently acquired neo-X. These results are generally in line with our theoretical predictions of the maladaptive evolution of young X chromosomes and accompanying demasculinization, but work in various other systems is needed to understand how universal this pattern is and how much of a contribution it makes to X-chromosome evolution.

In particular, one question that we did not address here is the time frame over which maladaptive evolution occurs. In the early stages of sex chromosome differentiation, the majority of the ancestral gene content on the Y chromosome will still be functional, and corresponding X-linked loci will be diploid and evolve under reduced selective efficacy. The accelerated rate of pseudogenization on the X will slow down as the Y chromosome degenerates and more X-linked loci become hemizygous, causing a shift in evolutionary dynamics to standard “faster-X.” Theory predicts that Y chromosomes first degenerate quickly after the recombination suppression but after they have lost about half of the gene content the process slows down, since the rates of degeneration by Muller’s ratchet, background selection, and genetic hitchhiking correlate with the number of active genes (Bachtrog, 2008). For instance, more than half of the genes on the *Drosophila miranda* neo-Y chromosome have been lost in a little over 1 million years, and the 15-million-year-old neo-Y of *D. pseudoobscura* is already highly degenerated (reviewed in Charlesworth, 2021). However, it is unclear if the neo-sex chromosomes of Drosophila, which have quickly co-opted a preexisting mechanism of dosage compensation, are representative of typical dynamics of Y degeneration, and much slower Y degeneration has been described in other systems (Charlesworth, 2021; Li et al., 2021). Nonrecombining regions with intermediate or low levels of Y/W degeneration have been described in various taxa (Charlesworth, 2021), e.g.: schistosomes (Elkrewi et al., 2021), frogs (Furman & Evans, 2018), crustaceans (Elkrewi et al., 2022), birds (Liu et al., 2021), fish (Sardell et al., 2021), and plants (Veltsos et al., 2019). It is therefore clear that many young X-linked genes can remain diploid for substantial periods of time.

The existence of a period of maladaptive evolution has implications for young homomorphic X chromosomes, but may also contribute to patterns observed on older sex chromosomes. The fact that X-linked genes with male-specific functions should be more prone to early maladaptive evolution suggests that the “demasculinization” that is observed on differentiated X-chromosomes of various species (Gurbich & Bachtrog, 2008) may begin before the degeneration of the Y. The subsequent shift in evolutionary dynamics could contribute to the differences in sex-biased gene contents of X chromosomes, which are for instance masculinized in mammals and demasculinized in *Drosophila* lineage (Gurbich & Bachtrog, 2008). The importance of temporal dynamics to the process of demasculinization is well appreciated in Drosophila, where only ancient male-biased genes are depleted from the X chromosome, whereas newly evolved ones are enriched on it (Zhang et al., 2010). While this potentially supports a role of diploid X evolution, the explanations brought forward to explain it typically assume a hemizygous X. Furthermore, Engelstaeder (2008) used modeling to show that the accumulation of deleterious mutations on diploid X-linked loci can slow down the degeneration of their Y-linked loci. An illustration of this is *PRSSLY*, a gene that was lost from the mammalian X chromosome in eutherians but retained on the Y (Hughes et al., 2022). If genes that function primarily in males are the ones that tend to accumulate deleterious mutations on a young X, this may lead to their preferential maintenance on degenerating Y chromosomes. The preservation of genes with male-biased expression on the Y has been observed (Crowson et al., 2017; Kaiser et al., 2011; Mahajan & Bachtrog, 2017; Zhou & Bachtrog, 2012) and is usually assumed to be driven by male-specific selection on Y-linked genes. In fact, degeneration of male-important genes on the X may drive their conservation on the Y, as well as the other way around.

Other peculiarities of X chromosomes may first arise early in their evolution. Repetitive sequences and transposable elements are overrepresented on X chromosomes as well as Y chromosomes (Bellott et al., 2010). The reduction in the effective population size of young X chromosomes (due to their lower population size, and potentially further exacerbated because they do not recombine in males) may already contribute to the accumulation of repeats. Finally, X-chromosomes are central to the two “rules of speciation”: Haldane’s rule (hybrid sterility or inviability tends to affect the heterogametic sex more than the homogametic sex), and the “large-X effect” (an excessive proportion of hybrid sterility loci maps to X chromosomes). Most of the clades that obey these rules have differentiated sex chromosomes, and explanations have typically invoked the faster-X hypothesis (along with other models) (Dufresnes & Crochet, 2022). However, hybridization patterns in *Aedes* mosquitoes, which have undifferentiated sex chromosomes, follow Haldane’s rule (Presgraves & Orr, 1998). Similarly, Dufresne et al. (2016) detected an excessive role of an undifferentiated X-chromosome in the reproductive isolation of tree frogs, and these rules have been suggested to apply to some clades with homomorphic sex chromosomes (Filatov, 2018). An excessive accumulation of deleterious mutations on homomorphic X chromosomes may provide an explanation for a large-X effect in this context, if compensatory mutations arise elsewhere in the genome. Similarly, if Y-degradation and X-sheltering affect different genes in close species, a mismatch between them may contribute to male sterility in hybrids. Studies of hybrids over a wide range of sex chromosome differentiation will in the future allow us to quantify the temporal dynamics of the large-X effect and Haldane’s rule, and the contribution of diploid X evolution.

In short, X-linked loci are expected to undergo a period of maladaptive evolution and demasculinization in the early stages of their differentiation. Our results show that contrary to what is often assumed, the peculiar evolutionary patterns on the X chromosome may arise before substantial degeneration of the Y has occurred and provide a novel framework for interpreting the increasing amount of data available for clades with young sex chromosomes.

## Supplementary material

Supplementary material is available online at *Evolution Letters* (https://academic.oup.com/evlett/qrac004)

qrac004_suppl_Supplementary_Material

## Data Availability

No data was created in this project.
