# Supplementary material for: Slower-X: reduced efficiency of selection in the early stages of X chromosome evolution
Source: Evol Lett. 2023 Jan 31;7(1):4–12. doi: 10.1093/evlett/qrac004 (PMC10091493; doi:10.1093/evlett/qrac004)
Supplement: qrac004_suppl_Supplementary_Material [file qrac004_suppl_supplementary_material.pdf]

Supplementary material and methods

Individual-based simulations

To confirm our inference of fixation probabilities and resulting substitution rates using diffusion approximation, we ran individual-based forward Wright-Fisher simulations, using the simulation package SLiM, version 3.6 (Haller and Messer, 2019). We simulated three cases: autosomal loci, hemizygous X-linked loci and diploid X-linked loci. We simulated 100 diploid individuals with separate sexes and an equal sex ratio. Each simulated genome had 100 loci. In generation zero, all the individuals are homozygous (or hemizygous) at all the loci for ancestral alleles and have the same fitness. Beneficial mutations with selective effects given in Table 1, occur at a rate of 10^{-6} per generation per locus and deleterious mutations occur at rate 10^{-5} per generation per locus. When simulating sex chromosomes, mutations were set to occur only on the X chromosome to represent the diploid X-linked locus case from Table 1., where the corresponding locus on the Y is fixed for an ancestral allele, while when simulating hemizygous X-linked loci, the Y chromosome was assumed to be empty/degenerated. Every individual contributes to the gamete pool of the following generation with the probability proportional to individual fitness. Individual fitness can be calculated as the product of the fitness effects of all the loci. The recombination rate was set to 0.5 to simulate free recombination between the loci. There was no recombination between the X and Y chromosomes. Each simulation was run for 1005000 generations, and the number of substitutions in the last 1 million generations was tracked. We only counted the substitutions occurring in the last 1000000 generations as it takes approximately $4N_e$ for a mutation to fix, so it

takes approximately 400 generations to reach a steady substitution rate in the simulation, and we filtered the first 5000 to be on the safe side. We ran separate simulations for different values of selection coefficient, *s*, corresponding to beneficial and deleterious mutations, and a range of dominance coefficients, *h*. For each of the cases, we ran 100 simulation replicates. The simulation code is available at https://github.com/andrea-mrnjavac/X-chromosome-theory/blob/main/README.md.

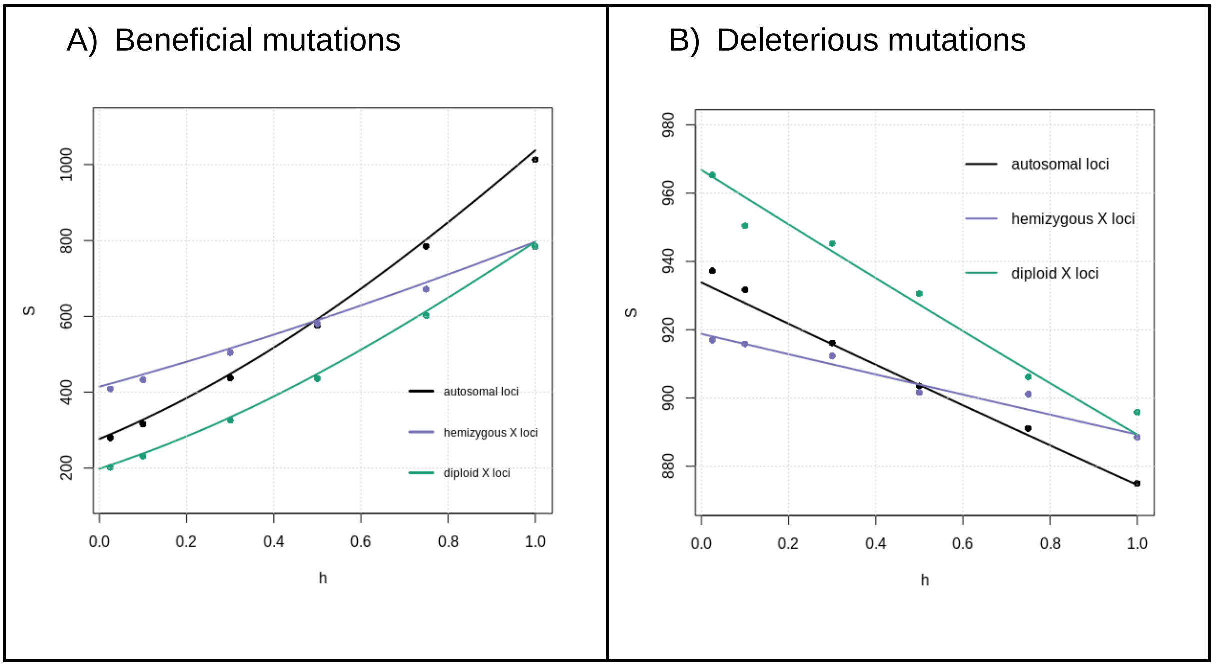

S.Figure 1 The mean number of beneficial (A) and deleterious (B) substitutions in simulations, for 100 loci in 1000000 generations, as a function of dominance coefficient *h*, for autosomal, hemizygous X-linked loci and diploid X-linked loci (points), plotted along the theoretical predictions (lines) for the number of substitutions with corresponding parameters. For beneficial mutations $N_{eA}s=3$, and for deleterious $N_{eA}s=-0.1$. The maximum percentage error between the theoretical predictions and simulation replicate means is 3.5% for autosomal loci, 2.9% for diploid X loci and 3.4% for hemizygous X loci, for beneficial mutations; and 0.5% for

autosomal loci, 0.9% for diploid X loci and 0.5% for hemizygous X loci, for deleterious mutations.

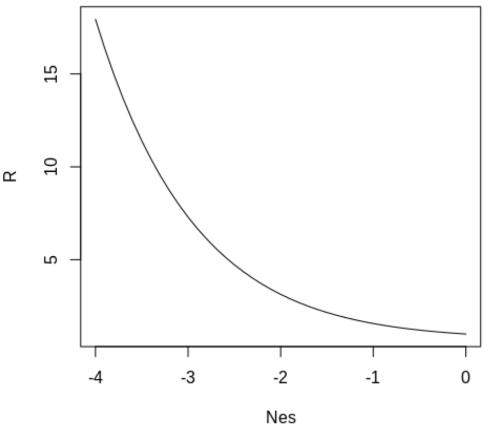

S.Figure 2. Ratio of substitution rates (R) at diploid X loci compared to autosomal loci, for recessive (h=0.1), deleterious mutations, as a function of selective effect of a mutation $N_e s$. The ratio exponentially increases as deleterious effect increases.

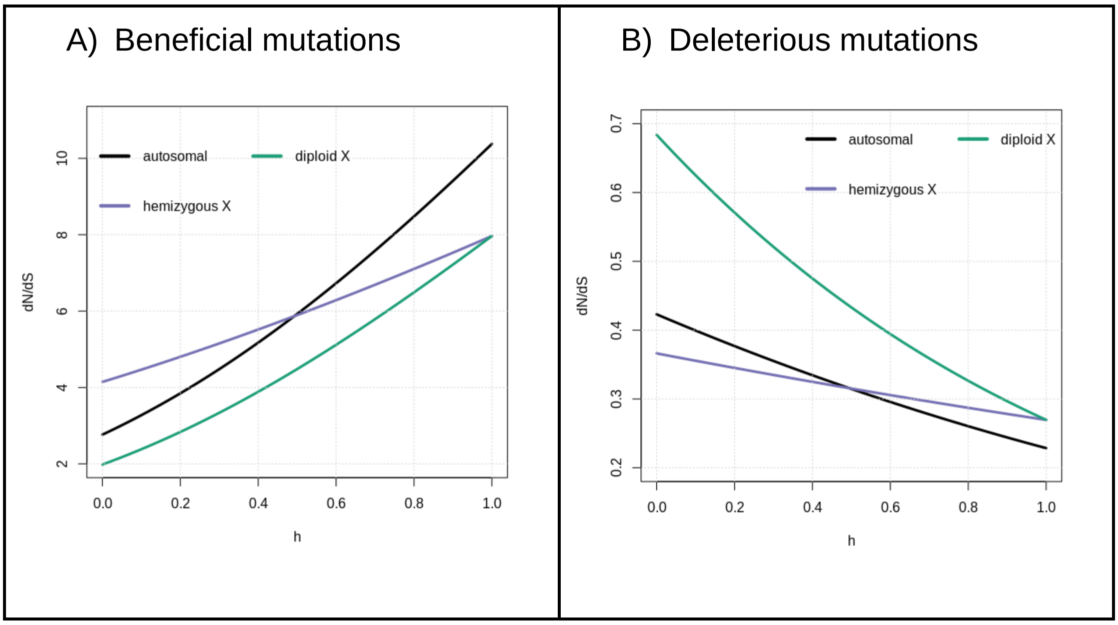

S.Figure 3. Expected dN/dS values for beneficial ($N_{eA}s$=3) and deleterious ($N_{eA}s$=-1) mutations for diploid X, hemizygous X and autosomal loci.

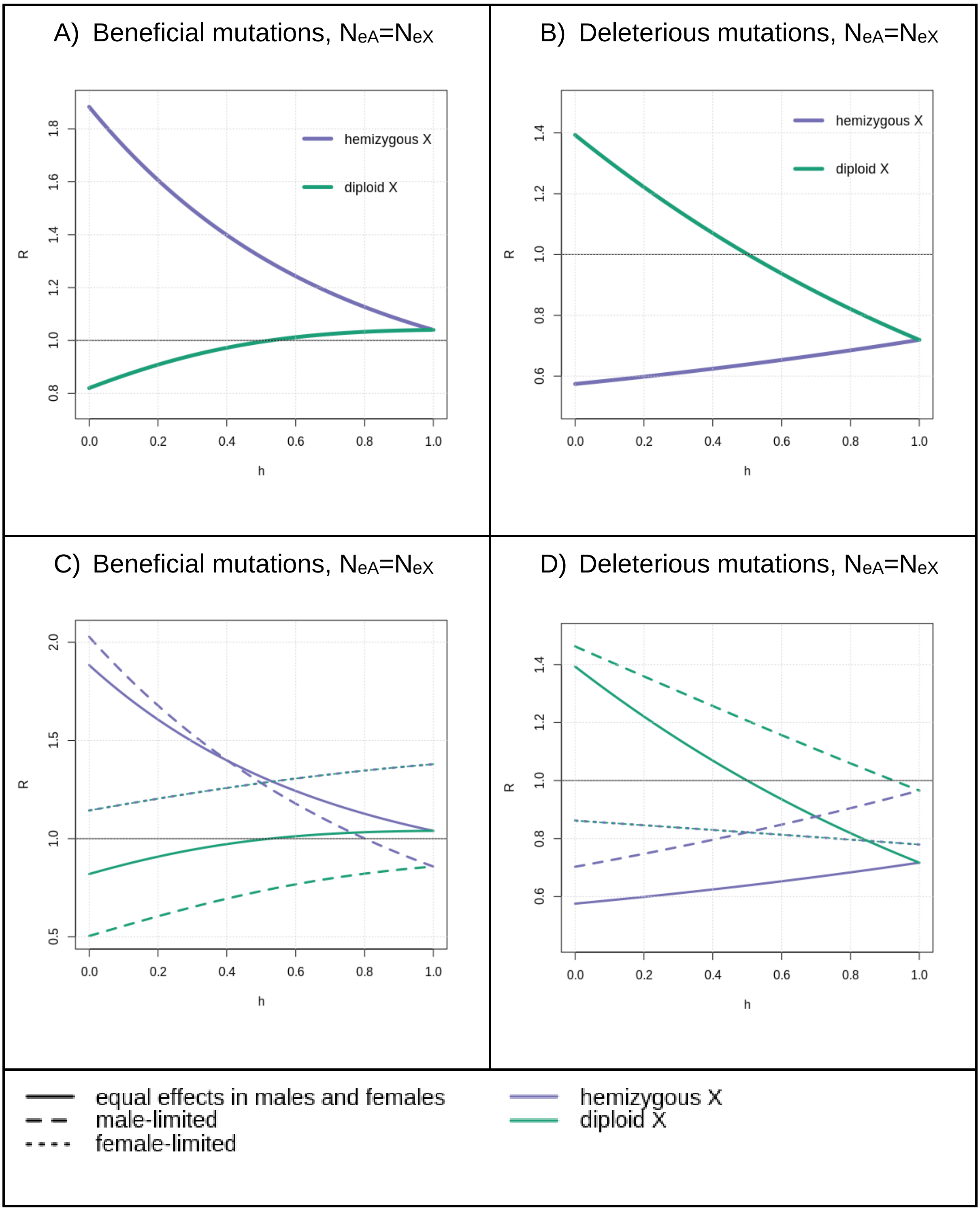

S.Figure 4. Ratios of substitution rates (*R*) on hemizygous X to autosomal loci and diploid X to autosomal loci as functions of dominance coefficient *h*, for beneficial, $N_{eA}s$= 3 (A and C) and deleterious, $N_{eA}s$= -1 mutations (B and D), where N_{eX}=N_{eA}. Mutations with equal effects in males and females are plotted in A and B, and in addition, in C and D, male-specific and female-specific mutations were plotted as

well. Here we can disentangle the effect of sheltering and the effect of reduced N_e, and fitness effects in males and females separately on the X/A ratio of substitution rates.
